# Food Composition and Dedicated Databases: Key Tools for Human Health and Public Nutrition

**DOI:** 10.3390/nu13114003

**Published:** 2021-11-10

**Authors:** Alessandra Durazzo, Massimo Lucarini

**Affiliations:** CREA—Research Centre for Food and Nutrition, Via Ardeatina 546, 01178 Rome, Italy

To better understand nutrition, food chemistry, and medicine, it is important to investigate biologically active constituents, which requires a detailed knowledge and coverage of the composition of compounds of nutritional and nutraceutical character. The categorization of substances and thus the implementation of specific and dedicated databases have now emerged, based on both analytical data and collected data derived from the literature through a standardized and harmonized approach [1].

Food composition databases aim to produce, collect, and present data in a standardized format to “speak a common language”, which allows the comparison of data from different national databases to foster an exchange and collaboration between countries [2,3]. Simultaneously, research is focused on the development of databases and models on metabolites in humans and novel dietary biomarkers [4,5,6].

The development of databases of nutrients, bioactive compounds, metabolites and dietary sypplements are key tools for human health and public nutrition and represent resources for a broad range of applications in different fields, i.e. food, nutraceutical, pharmaceutical, epidemiology and medicinal areas [7,8,9,10,11,12].

The initial construction of a dataset of specific nutrients, bioactive compounds, or bioactive compounds’ class and their inclusion in a specified and standardized database should be monitored. Moreover, an update and expansion of the database for a more comprehensive source of data and information is encouraged. Databases dedicated to particular and characteristic categories of foods are also promoted: traditional, certified, and recipe databases [13,14,15,16].

Hoteit el al. [17], aiming at studying non-conjugated-industrially-produced-trans fatty in Lebanese foods, especially regarding Elaidic acid and Linolelaidic acid, monitored 145 food samples consisting of 3 categories: traditional dishes, Arabic sweets, and market food products. The results showed that approximately 93% of the products tested in Lebanon, between 2019 and 2021, met the World Health Organization recommendations, while approximately 7% exceeded the limit [17].

Beltrá et al. [18] studied sodium content of foods sold in the Spanish market, as results of the BADALI Project. Balakrishna et al. [19] identified the nutrient patterns in South African foods to support the National Nutrition Guidelines and Policies. Marcotrigiano et al. [20] reported the results obtained from a field investigation on nutritional and hygienic features in the Apulia region (Southern Italy) as an integrated control plan in primary schools.

First and foremost, the design and construction of food databases require the exact identification of foods from an adequate food nomenclature and a precise description of the foods. There is a general consensus on the importance of the nomenclature, description, and classification of foods and food groups. A coherent food description system is essential for comparing and/or exchanging data from different databases, and the data of the same nature from different organizations and countries. Moreover, matching procedures for linking different databases should be encouraged [21].

Food composition and other dedicated databases, as well as metabolomic databases and biomarker repositories, represent a unique data resource for nutritionists, dietitians, and researchers for several applications, i.e., dietary assessments, exposure studies, food labeling, epidemiological studies, and clinical trials. Concerning dietary assessment, Witkowska et al. [22] reported the assessment of plant sterols in the diet of adult polish population with the use of a newly developed database. Regarding food labeling, Castro et al. [23] reported the comparison of healthiness, labeling, and price between private and branded label packaged foods in New Zealand (2015–2019).

Applications and the utilization of databases from nutrition- and medicine-related fields in other contexts are explored, and current research trends are defined. Delgado et al. [24] described the usefulness and limitations of food databases with particular emphasis what concerns sustainable diets, the food ‘matrix effect’, missing compounds, safe processing, and in guiding innovation in foods, as well as in shaping consumers’ perceptions and food choices.

## Data Availability

Not applicable.
